# Design and implementation of a low-cost gimbal-based angular ultrasound gantry for optimal tissue slice selection using deep learning

**DOI:** 10.1016/j.ohx.2025.e00676

**Published:** 2025-07-19

**Authors:** Abhishek Kumar, Akshay S. Menon, Divyansh Sharma, Raviteja Sista, Debdoot Sheet

**Affiliations:** Indian Institute of Technology Kharagpur, West Bengal, 721302, India

**Keywords:** Automated gantry scanner, Excised tumor analysis, Optimal slice selection, Ultrasound imaging

## Abstract

Ultrasound (US) is a widely popular imaging technique for the diagnosis of tumors and associated soft tissue pathology. Traditionally, excised tumor masses are manually sliced for microscopic examination, which is a resource-intensive, time-consuming process, and prone to human error. The proposed work addresses these challenges by developing a cost-effective US gantry system integrated with a deep learning algorithm to automate the tissue slice selection process. This system scans the entire tumor and by integrating a deep learning algorithm predicts the optimal slice to assist its preparation for microscopic analysis. Automating this process reduces the time and resources required while minimizing the risk of human error. Optimal tissue slice reduces sampling associated uncertainty in diagnosis and treatment planning. Thereby determining tumor grade and type, and helping to reduce the treatment risks. The initial development focused on a linear US gantry that moves in one direction to acquire B-mode images. However, this design is limited, as it cannot fully capture the tumor’s structural complexity. In order to overcome this, we developed an angular US gantry that can maneuver along multiple angles, acquiring a broader range of images for comprehensive geometric analysis. The angular gantry demonstrated significant improvement, achieving 98% accuracy in selecting the optimal tissue slice.

## Graphical abstract

**Figure:**
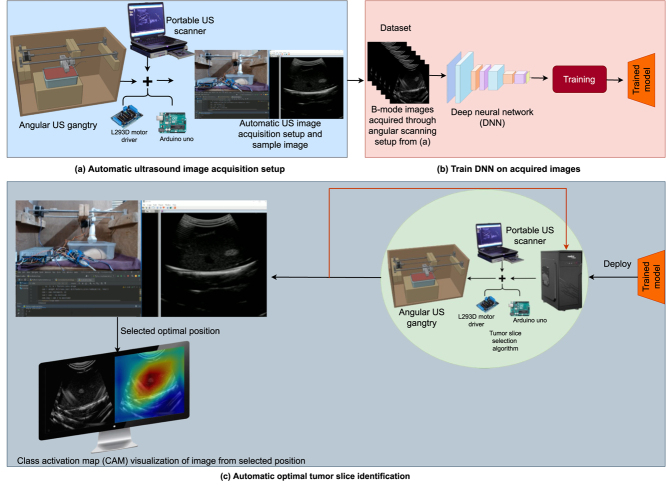

## Specifications table

**Table:** 

Hardware name	Angular ultrasound gantry scanner

Subject area	•Engineering • Medical science • Educational tools and open source alternatives to existing infrastructure

Hardware type	•Educational purpose • Automated optimal tumor slice position selection • Assist pathologist in tumor analysis

Closest commercial analog	Automated acquisition of ultrasound images using robots

Open source license	CC BY-NC 4.0

Cost of hardware	$ 88.3 USD

Source file repository	https://osf.io/k8cge/files/osfstorage

## 1 Hardware in context

**Fig. 1:**
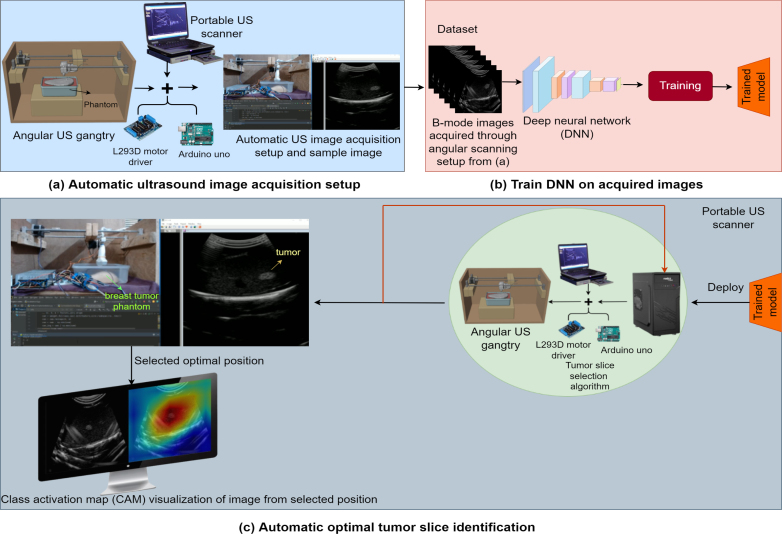
The pipeline of angular US gantry for selecting the optimal tissue slice and its location.

Ultrasound (US) is a non-invasive imaging technique widely used for real-time visualization of internal organs and is crucial in applications such as tumor diagnosis, abnormality detection, and fetal monitoring [1], [2], [3]. US plays a significant role in tumor biopsies, particularly during excision, where a tumor mass is surgically removed for detailed examination [4]. Traditionally, pathologists rely on manual expertise to select tissue slices for microscopic analysis, a procedure that, while effective, is often time-consuming, resource-intensive, and prone to human error [5], [6]. These limitations can lead to diagnostic inconsistencies, highlighting the need for automation. To address these challenges, a low-cost angular US gantry has been designed and developed. This hardware facilitates precise scanning of subjects from various angles, offering a more comprehensive view than traditional methods. Unlike automatic robotic systems, which can be costly and complex to operate, the angular US gantry with 3 degrees of freedom (DOF) offers an accessible alternative [7], [8], [9]. It maintains the precision needed for accurate imaging while minimizing the operational complexity, making it an ideal choice for diagnostic centers, institutions, research labs with limited budgets or those focused on efficient diagnostics. The deep learning (DL) based algorithms enable to automatically scan the entire subject, analyze tissue patterns, and predict the optimal slice location that needs to be excised for microscopic examination [10], [11], [12]. This reduces the reliance on manual selection, significantly improving the speed and accuracy of tumor analysis. Building upon our previous work on linear design [13], the angular gantry’s capability to perform both linear and angular movements ensures a thorough examination of the tumor, enabling optimal slice selection with greater precision. By automating this process, the angular US gantry assists pathologists in reducing human error, streamlining the workflow, and improving the quality of diagnostic outcomes. Its low-cost design makes it suitable for use remotely or in resource-constrained settings, offering a practical solution for enhancing diagnostic accuracy in medical and research environments. The pipeline for predicting optimal tumor slices using the angular US gantry is illustrated in Fig. 1.

## 2 Hardware description

**Fig. 2:**
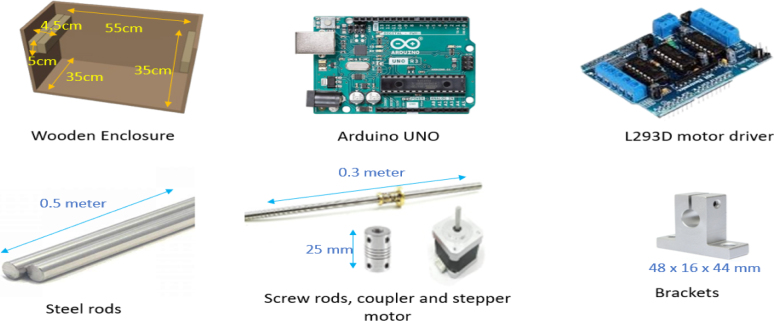
Main components used in the creation of the angular US gantry.

The low-cost angular US gantry scanner operates through a carefully designed assembly of components that enable linear and angular movement. As illustrated in Fig. 2, the gantry’s functionality is optimized by the seamless coordination of its hardware components. The specifications for all components are provided in Table 1, and the details of each hardware component is given below.

### 2.1 Wooden enclosure

The wooden enclosure provides a stable and cost-effective base for the gantry scanner. Its natural vibration-absorbing properties contribute to the system’s overall stability during operation [14]. The dimension of the wooden enclosure is 55 cm in length, 35 cm width, and 35 cm height.

### 2.2 Stepper motor

The National Electrical Manufacturers Association (NEMA)-17 stepper motors^1^ are integral to moving the gantry header linearly by the total number of steps ($n_{steps}$), including each linear step size ($s_{lin}$). Their precise control allows for accurate positioning of the gantry, ensuring reliable movement along the desired axis.

**Table 1:** Specifications of the components of angular US gantry.

Component	Specifications
	- Material: Wood
1. Wooden enclosure	- Dimensions: 55cm×35cm×35cm

	- Model: NEMA 17
	- Step Angle: 1.8°(200 steps per revolution)
2. Stepper motor	- Voltage: 12 V
	- Torque: 4.2 kg-cm

	- Model: SG series
3. Servo motors	- Motor used: 2
	- Voltage: 7.2 V
	- Torque: 1.2 kg-cm

	- Steel rods: 2
	- Length: 0.5 m
4. Steel and screw rods	- Screw rod: 1 (type: trapezoidal (TR8 × 2), pitch: 2 mm)
	- Length: 0.3 m
	- Outer Diameter: 8mm

	- Model: SK8
5.Coupling and brackets	- Brackets fit for shaft diameter.: 8mm (material: Aluminium)
	- Coupling shaft diameter: 8 mm

	- Microcontroller: ATmega328P
	- Operating voltage: 5 V
	- Digital I/O pins: 14 (6 PWM)
6. Arduino UNO	- Analog input pins: 6
	- Memory: 32 kB flash, 2 KB SRAM, 1 kB EEPROM
	- Clock speed: 16 MHz
	- Logic control voltage: 4.5 V to 5.5 V

	- Continuous output current: 1.2 A
7. L293D motor driver shield	- Motor control: 2 servo motors and 1 stepper motor (In this work)
	- Functionality: Controls motor direction and speed
	- Material and model: Poly lactic acid (PLA) and 3D model

8. Angular gantry header	- Structure: Integrates four 3D models for gimbal setup (a) Probe holder dimension: 4.9cm×2.5cm×2.0cm
	(b) Two servo motor bedding dimension: 4.0cm×2.5cm×2.5cm
	(c) Connector to probe holder and servo motor dimension: 3.0cm×1.5cm×0.9cm

	- US scanner: Ethiroli Tiny 16a portable scanner
9. US scanner and phantom	- Probe: 64-element convex probe (C360)
	- Phantom: CAE commercial breast phantom containing a tumor and a cyst

### 2.3 Servo motor

Angular gantry header utilizes two servo motors^2^ to control the angular movements ($s_{\theta }$) including the pitch angle ($\theta_{max}$) and roll angle ($\phi_{max}$)movements. This dynamic adjustment capability enables the gantry to acquire images from multiple perspectives, enhancing the ability to precisely scan the subject from various angles.

### 2.4 Steel and screw rods

The steel^3^ and screw rods^4^ are utilized to maintain structural integrity and facilitate the linear movement of the gantry header. The rods ensure stability during scanning, while the screw rod aids in the forward and backward movement of the header.

### 2.5 Coupling, brackets and screw

Coupling^5^ mechanisms connect the stepper motor to the rods, allowing for efficient power transmission. They also compensate for misalignments, reduce vibrations, and simplify assembly and maintenance, ensuring smooth operation. The brackets^6^ are used to secure the steel rods to the wooden enclosure at both ends, providing support for the angular US header’s movement, and they are fixed in place with screws.

### 2.6 Arduino UNO

The Arduino UNO^7^ [15] serves as the microcontroller that regulates the entire system. It controls the direction of each gimbal movement, including the linear movements of the stepper motor ($n_{steps}$) and the angular movement of the servo motor ($s_{\theta }$), enabling coordinated linear and angular adjustments of the US gantry header.

### 2.7 L293D motor controller driver shield

The L293D motor driver shield^8^ controls servo and stepper motors using an H-bridge configuration,^9^ which enables bidirectional DC motor control by switching voltage polarity. This allows precise clockwise and counterclockwise rotation required for the scanning process.

### 2.8 Angular gantry header

**Fig. 3:**
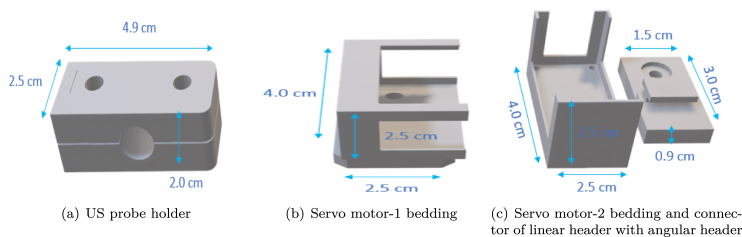
3D printed components of the gantry header are shown in figure (a) holds the US probe, (b),(c) are the beddings for the servo motor and connector of the linear header with an angular header.

**Fig. 4:**
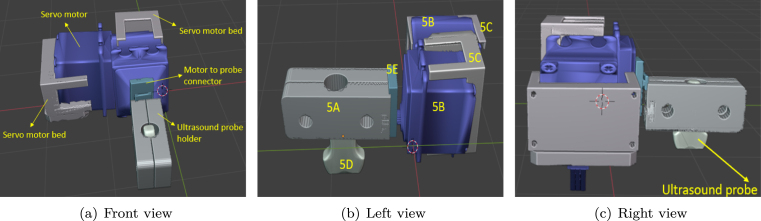
The figure shows the different views of the angular gantry header (a) represents the front view of the gantry header, (b) and (c) shows the header from the left and right view, respectively.

Our linear gantry header integrates four 3D model^10^ structures to form the complete angular US gantry, which resembles a gimbal-like setup. Fig. 3(a) shows the US probe holder, while Fig. 3(b) displays the mounting frame for the first servo motor. Fig. 3(c) illustrates the mounting frame for the second servo motor and the support structure connecting the servo motors with the US probe. By combining these four models with the servo motors, the angular movement of the US gantry scanner is achieved. The image of the complete angular gantry header from different views are presented in Fig. 4.

### 2.9 US scanner and phantom

**Fig. 5:**
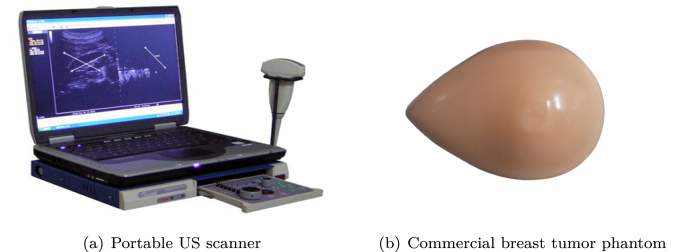
Figure (a) shows the Ethiroli Tiny16a portable US scanner used in the experiment, while (b) depicts the commercial breast phantom with a tumor used in our experiment.

In our experimental setup, we used an Ethiroli Tiny 16a portable US scanner^11^ (Fig. 5(a)) equipped with a 64-element convex US probe (C360) for scanning. Additionally, we used a CAE commercial breast phantom^12^ (Fig. 5(b)) containing tumor as the scanning object for our experiment.

## 3 Design files summary

The hardware design and its details are shown in Table 2, where the link in the table will redirect to the zip file containing the various components of the angular gantry. The code for the automated angular US gantry and its implementation is available at https://github.com/abhi1611/Gantry-scanner.

**Table 2:** Details of design files including filenames, file types, open source licenses, and file locations.

Design filename		File type	Open source license	Location of the file
Gantry	• Wooden enclosure • Linear gantry header • Ultrasound probe • Steel rod • Screw rod • Brackets • Stepper motor	CAD	CC BY-NC 4.0	https://osf.io/m4dx7

Angular gantry header	• Ultrasound probe holder • Servo motor bedding • Motor to probe connector	CAD	CC BY-NC 4.0	https://osf.io/h6rj3

## 4 Bill of materials summary

The billing details of components of angular US gantry are shown here: https://osf.io/xqkfz.

## 5 Build instructions

### 5.1 Mechanical assembly of the angular gantry

• Fix two parallel steel rods (2A) onto a wooden enclosure (9) using brackets (1) and screws (10).
• Position the linear gantry header (6) between the steel rods to enable smooth sliding motion.
• Insert a screw rod (2B) into one of the holes in the linear gantry header.
• Connect one end of the screw rod to a stepper motor (4).
• Control the stepper motor using an Arduino UNO board in combination with an L293D motor driver.
• Attach the gimbal-based angular header (5) of the US gantry below the linear header.
• Mount the US probe (5D) of the portable device onto the angular gantry header.

**Fig. 6:**
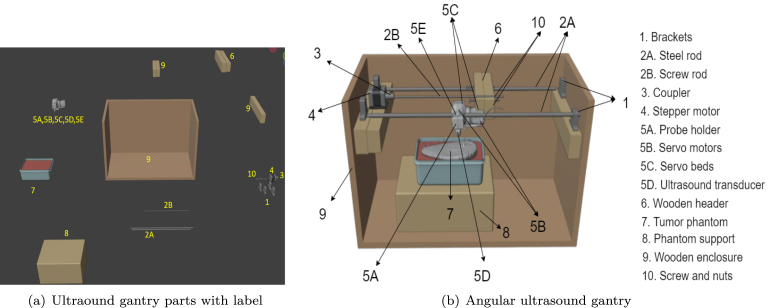
(a) shows the labeled components of the angular ultrasound gantry, and (b) shows the fully assembled angular ultrasound gantry with corresponding labels.

After completing the gantry assembly, a command is issued to the Arduino via a Python script. The L293D motor controller then rotates the stepper motor either clockwise or counterclockwise, causing the screw rod to move linearly in the forward or backward direction. At each linear step, the angular US header rotates the probe to fixed angles in various directions, thereby scanning the subject and capturing US images between consecutive linear movements. Assembly and operational videos are included in the supplementary material, and the assembly of the angular gantry is shown in Fig. 6.

### 5.2 Wire connection

The Angular US gantry system is powered and controlled by an Arduino UNO board, equipped with an L293D motor driver shield to manage the movement of both stepper and servo motors. This setup allows for precise linear and angular motions, making the gantry capable of scanning the subject efficiently. The wiring configuration is carefully designed to ensure that the motors receive the appropriate signals and power for the desired movements. The stepper motor, which controls the linear movement of the gantry, has four wires that connect to the L293D motor driver shield. In this setup, two pairs of wires from the stepper motor are connected to the motor driver shield’s M1 and M2 ports. The stepper motor controls the linear movement, allowing the gantry to move forward and backward precisely. The L293D motor driver, acting as an intermediary between the Arduino UNO and the stepper motor, regulates the current and ensures that the motor receives the correct sequence of signals for each step. This enables smooth and accurate linear motion.

**Fig. 7:**
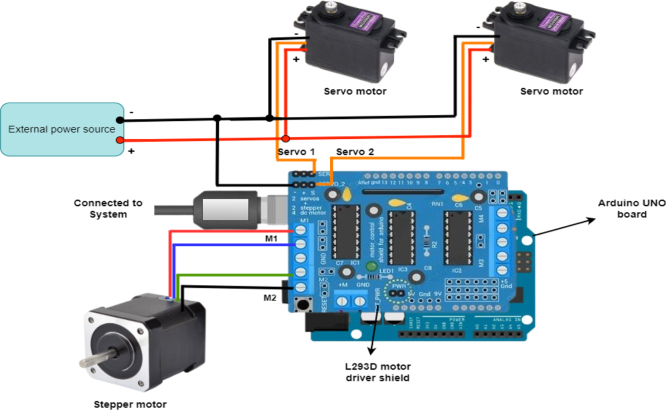
The diagram of the motor wiring connections and illustrates how the Arduino interfaces with the L293D motor driver.

In addition to the stepper motor, two servo motors are used with the angular gantry header to control its angular movements, specifically the pitch and roll angles. Each servo motor has three wires: power, ground, and signal. The power and ground wires supply the necessary voltage to the servo motors, while the signal wire communicates the movement instructions from the Arduino. In this system, the primary or signal wire of the first servo motor is connected to the “servo 1” port of the L293D motor driver shield, and the signal wire of the second servo motor is connected to the “servo 2” port. These ports control the angular position of the US gantry, allowing it to rotate along the pitch and roll axes. By manipulating the signals sent to the servo motors, the system can adjust the angular position of the US probe to acquire images from different orientations. An external power source^13^ is used to power the system to ensure that the servo motors receive sufficient power. The negative terminal of the power source is connected to the negative terminals of both servo motors, ensuring a common ground for the power supply. The positive terminal of the power source is connected to the positive terminals of both servo motors, providing them with the required operating voltage. To maintain electrical consistency and ensure the correct functioning of the motor driver, a connection is also made between the negative terminal of the external power source and the negative terminal of the L293D motor driver. The Arduino UNO board is connected to the personal computer with USB port. The overall wire connection flow diagram in Fig. 7.

### 5.3 Control algorithm

The proposed method employs a combination of linear and angular scanning to determine the optimal tumor slice for microscopic analysis using an US gantry. We conducted experiments using multiple deep learning networks, including AlexNet [16], VGG16, VGG19 [17], ResNet18, ResNet50 [18], MobileNet [19], DenseNet [20], ConvNeXt [21], DeiT [22], and Swin Transformer [23], to evaluate their performance in tumor detection and classification. The Swin Transformer demonstrated the best results among these models, offering superior accuracy and robustness in identifying optimal tissue slices. The algorithm systematically explores different positions and orientations of the US transducer and evaluates each scanned position based on the probability of tumor presence. The final goal is to identify the position ($L_{max}$) with the highest probability ($P_{max}$), ensuring that the most representative slice is selected for further microscopic examination.

The scanning process involves two key components: angular and linear movement, as described in Algorithms 1 and 2. The linear movement is defined by step size $s_{\text{lin}}$, determining how far the US transducer moves in each step. The coordinates $l_{x}$ and $l_{y}$ represent the current position of the transducer along the linear trajectory. An US image is captured at each linear position, and the trained Swin Transformer model predicts a probability score ($l_{\text{prob}}$) indicating the likelihood of tumor presence. This process continues for $n_{\text{step}}$ iterations, where $n_{\text{step}}$ represents the total number of linear movements. This ensures that the scanning covers a broad search space before refining the search further. After each linear step, angular scan is performed by rotating the transducer at different angles, where the step size is defined by $s_{\theta }$, which includes the pitch ($\theta_{max}$) and roll ($\phi_{max}$) angles. The angular movement systematically sweeps through a set of orientations ($\theta_{max},\phi_{max}$), adjusting the scanning direction based on the iteration count. The angular search is governed by two primary range variables, P and R, representing the pitch and roll angle ranges, respectively. If the direction indicator d is 1, then P is set to $range\left(-s_{\theta },s_{\theta }+1\right)$, ensuring that the pitch angles sweep symmetrically around the current position. Otherwise, if d is 0, the range is reversed to $range\left(s_{\theta },-s_{\theta }-1\right)$, ensuring an alternate scanning pattern. This structured alternation allows for a systematic exploration of angular positions.

During the angular scanning process, for each value i in P, the secondary range R ensures a zigzag pattern in angular scanning, covering all relevant orientations effectively. The angular positions are then calculated using a scaling factor α ($pos_{i}=\alpha \times i$, $pos_{j}=\alpha \times j$). The parameter α is a crucial factor in determining the precision of angular movements. It defines the granularity of angle changes, typically set to 2° per step, ensuring that each angular increment contributes meaningfully to the scan coverage. The values $pos_{i}$ and $pos_{j}$ represent the angles at which the probe is positioned. Once the linear and angular scans are performed, the algorithm compares the probability values from both processes (Algorithm 2). The maximum probability values obtained from the linear and angular searches are denoted as $l_{\text{prob}}$ and $a_{\text{prob}}$, respectively. If the linear movement yields a higher probability, the best position is recorded as $\left(l_{x},l_{y}\right)$. Otherwise, if the angular movement results in a higher probability, the best orientation is stored as $\left(\theta_{\text{pos}},\phi_{\text{pos}}\right)$, and the associated probability is assigned to $P_{\text{max}}$. To evaluate the effectiveness of each scanned position, Class Activation Mapping (CAM) [24] is employed to visualize the regions within the US image that contribute most to the model’s decision. This visualization aids in confirming whether the detected tumor region is appropriately focused. The gantry header moves to the position where probability is maximum.

A critical aspect of the algorithm is the iterative refinement process (Algorithm 2), where the step size $s_{\text{lin}}$ is gradually reduced to ensure a finer search around the most promising locations. This refinement continues until the step size reduces below a predefined threshold ϵ, providing the search converges to the optimal slice location. The final output of the method is the optimal scanning position and orientation $\left(L_{\text{max1}},L_{\text{max2}}\right)$ along with the maximum probability $P_{\text{max}}$. This approach effectively balances coarse and fine search strategies, ensuring that the US gantry systematically selects the most relevant tumor slice for microscopic analysis.

**Figure:**
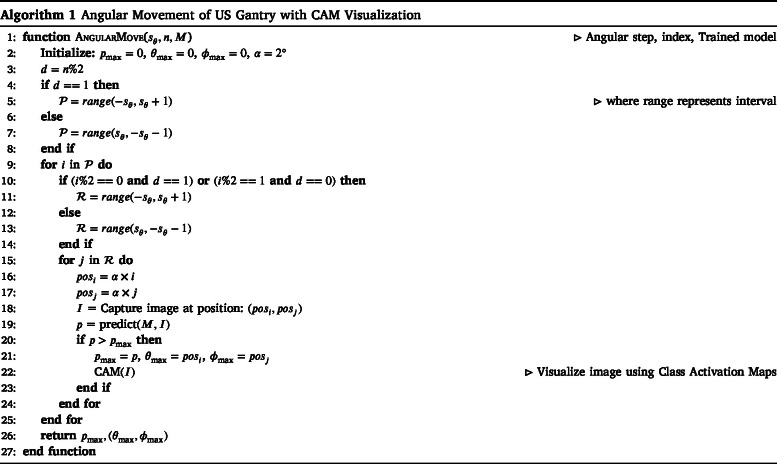

**Figure:**
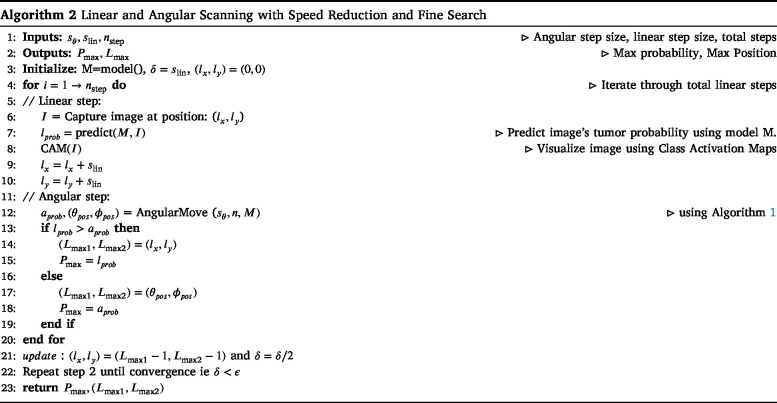

## 6 Operation instructions of the angular US gantry

The angular US gantry is a precision tool designed to scan tissue samples to assist pathologists in identifying optimal tissue slices for microscopic examination. This device combines automated movements with advanced imaging techniques, allowing efficient and accurate detection of areas most likely to contain significant tissue abnormalities. The gantry can perform linear and angular scans, capturing images at various positions to evaluate tissue characteristics. Below are the step-by-step instructions for operating the gantry system.

### 6.1 Step 1: Angular US gantry system setup

• Securely place the angular US gantry on a stable, flat surface.
• Ensure that all screws, brackets, and connections are tight and that the gantry structure is securely fastened.

### 6.2 Step 2: Software initialization

**Fig. 8:**
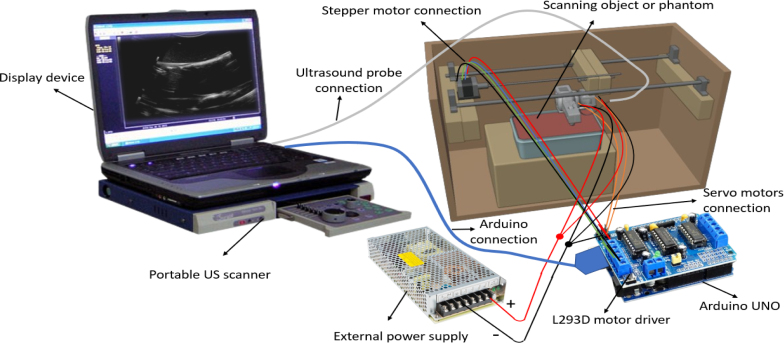
The setup of angular US gantry with visualization unit is shown in the figure.

• Connect the angular US gantry (Fig. 8) to the portable US scanner using the Ethiroli application and integrate it with Python to start the gantry.

### 6.3 Step 3: Linear movement of the gantry

• The Python script takes the input steps ($n_{steps}$) to control the linear movement of the gantry. The stepper motor rotates, driving the screw rod forward or backward, which enables the linear header to move along the steel rods.
• The gantry will move in small increments according to the predefined steps $s_{lin}$ according to Algorithm 2. After each linear step, the gimbal’s angular movement will adjust the probe’s angle, as discussed in Algorithm 1.

### 6.4 Step 4: Gimbal-based angular movement

The system will now perform angular movements of the US probe using Algorithm 1, which allows scanning at various $\theta_{max}$ and $\phi_{max}$ angles. After each linear step, the gimbal header adjusts the probe at fixed incremental angles (α), moving it through pitch and roll angles to scan the subject. Each angular step, incremented by α, allows the acquisition of a US image at every position, ensuring that all necessary angles are covered for a complete B-mode US scan.

### 6.5 Step 5: Data collection and optimal slice selection

**Fig. 9:**
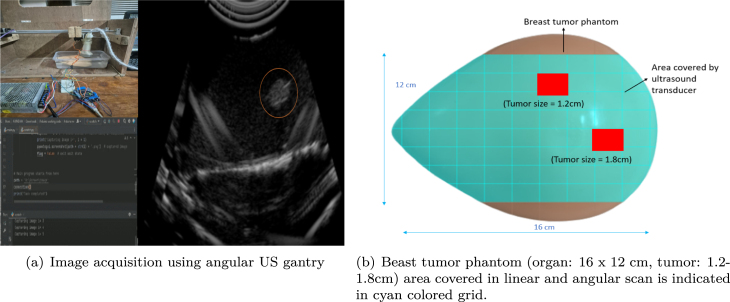
(a) Shows the setup for image acquisition using the angular US gantry with a CAE breast tumor phantom, where the tumor is highlighted in the red circle. (b) Illustrates the phantom area covered by the gantry during image acquisition and tumor slice prediction. (For interpretation of the references to color in this figure legend, the reader is referred to the web version of this article.)

**Fig. 10:**
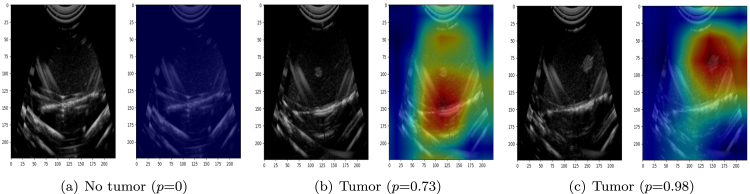
The given figure shows the results of angular US gantry. (a) shows the scanned image with prediction that has no tumor region. similarly (b) and (c) shows the real time scanned image and the prediction of tumor region with the help of CAM.

• As the gantry performs the linear and angular movements the US probe scans at different angles (Fig. 9(a)), the US images will be captured and the images will be saved in the local device.
• We trained the DNNs to detect and classify tumors using the acquired images. During inference, we tested the algorithm on different phantoms (Fig. 9(b)), where the angular US gantry scanned from multiple angles, computing the probability of predicting the optimal slice at each linear, pitch, and roll movement. Once all linear and angular movements were complete, a refinement scan was performed around the most promising locations using a reduced step size to enhance precision. The system then determined the best location ($L_{max}$) for the optimal scan and moved the gantry header to that position, as calculated in Algorithm 2. The maximum probability ($P_{max}$) was then returned, indicating the highest likelihood of accurately identifying the tumor slice. Class Activation Mapping (CAM) further validated the model’s decision, highlighting the key regions influencing the prediction. The final predicted slices and their probabilities are shown in Fig. 10.

## 7 Training and experiment

### 7.1 Dataset

To develop and validate the deep learning (DL) model for optimal US slice prediction, we curated a dataset comprising 4900 B-mode US images, encompassing both tumor and non-tumor slices. These were acquired using our custom-built gimbal-based US gantry capable of multi-angular scanning. The programmable design allowed stable, automated acquisition from varied orientations, mimicking real-world probe variability in clinical imaging [25], [26]. Scanning from different angles added variety in shape and structure to the dataset, helping the model to work better on different cases. Water immersion was used as a coupling medium to maintain consistent acoustic transmission and eliminate air gaps, ensuring reproducible, pressure-independent image acquisition.

Multiple commercial breast tumor phantoms were used in this study, selected for their acoustic similarity to human soft tissue properties [27], [28]. The breast phantom was measured 16 × 12 cm with embedded tumors ranging from 1.2 to 1.8 cm in diameter, representing a whole breast segment. We also tested custom PVA-based phantoms to evaluate the system’s adaptability to smaller anatomical structures, including a rat liver model [29].

### 7.2 Network training

The training pipeline was designed to use the angularly consistent data captured using fixed 2°steps along the pitch and yaw axes. This ensured structured spatial variation between consecutive slices and reduced the effect of frame misalignment. The system’s reliance on B-mode US was a conscious choice, driven by its real-time acquisition capability, hardware integration ease, and broad clinical availability. The system is intended to assist pathologists in clinical decision-making rather than replace them, functioning as a decision-support tool where trained pathologists verify all predictions to ensure diagnostic safety. We evaluated diverse model architectures, including conventional CNNs, efficient mobile-friendly models such as MobileNet, and transformer-based networks. All models were trained under a consistent hyperparameter configuration: a learning rate of 0.0001, batch size of 32, and 200 training epochs, using the Adam optimizer with cross-entropy loss as the objective function. The input image resolution was standardized to $R^{1\times 224\times 224}$ for CNN-based models and $R^{1\times 384\times 384}$ for transformer-based models. Before training, the images were preprocessed and augmented using a set of image-level transformations such as rotation, flipping, and normalization [30], [31]. These augmentations simulate variability in tissue structure and probe positioning, enhancing the model’s ability to generalize under diverse acquisition conditions. The training was conducted on an NVIDIA GTX1080 GPU running a Linux system. As shown in Table 3, MobileNet and other lightweight models demonstrated superior inference speeds and lower computational cost, while Table 4 shows that the Swin Transformer achieved the highest classification performance across accuracy, precision, recall, and F1-score metrics. Although the internal architectures of the models were not modified, each network underwent multiple training trials with varied hyperparameters. The best-performing configuration from these trials was selected for final evaluation. This comparative analysis highlighted key trade-offs between model accuracy and deployment feasibility, which will guide the integration of lightweight models in future hardware-constrained implementations. Following model training and hyperparameter optimization, we proceeded to evaluate performance on unseen phantom data. The inference pipeline predicts the most informative tissue slice by scoring each frame acquired during the angular scan. The prediction was based on the algorithm detailed in Algorithms 1 and 2, which compute class probabilities and select the frame with the maximum relevance score.

CAMs were generated to interpret and validate model decisions, overlaying heatmaps onto input images to highlight spatial regions that influenced the model’s predictions. This interpretability feature confirms that the model attends to diagnostically relevant tumor areas. These CAMs were visually compared against expert-marked annotations, demonstrating strong spatial alignment and validating the model’s ability to localize relevant tumor regions. Representative visual comparisons are shown in Fig. 14, supporting the model’s trustworthiness in clinical decision support.

**Table 3:** Comparison of model performance.

Model	Parameters	Flops	Throughput (fps)
AlexNet	61M	0.71G	265
VGG16	138M	15.52G	35
VGG19	143M	19.69G	45
ResNet18	11M	1.83G	203
ResNet50	25M	4.13G	83
MobileNet	3.5M	0.32G	85
DenseNet121	8M	2.9G	28
ConvNeXt	28M	4.49G	63
DeiT	87M	0.9T	16
Swin	88M	16G	25

The system’s real-time inference performance was evaluated using an Intel i7 processor and an NVIDIA GTX 1080 GPU. Inference tests were conducted using the Swin Transformer, which consistently achieved the highest accuracy. The Intel i7 processor achieved an average inference time of approximately 150 to 180ms per frame. In contrast, the GTX 1080 GPU significantly reduced the inference time to around 35 to 45ms per frame requirement for real-time deployment. This confirms that the integrated gantry and deep learning framework can process data on the fly without introducing latency that would disrupt clinical or intraoperative workflows. The pipeline combines automated multi-angle image acquisition with a carefully selected and optimized deep learning framework, enhanced by explainable predictions and real-time inference capability. The integration of these components supports accurate and interpretable tissue slice selection, with the potential for scalable deployment in pathology labs, surgical environments, and low-resource settings.

**Fig. 11:**
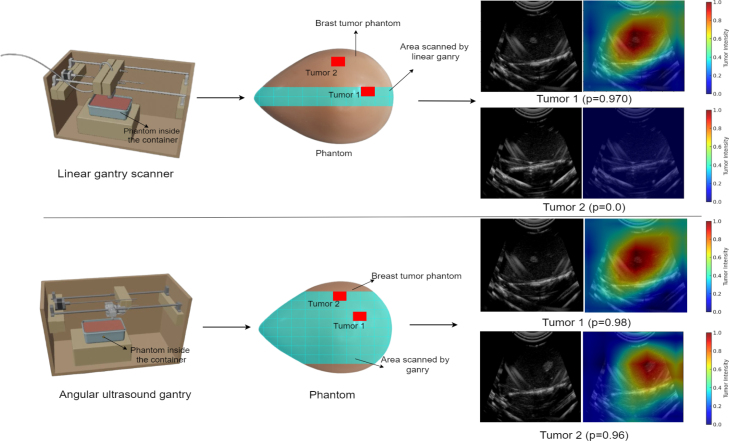
Comparison analysis on linear and angular US gantry scanner. (For interpretation of the references to color in this figure legend, the reader is referred to the web version of this article.)

## 8 Validation, and characterization

The gimbal-based US gantry was rigorously validated using a CAE Blue commercial breast phantoms engineered to mimic human tissue properties [32]. These phantoms simulate variable tumor placements and tissue densities, making them ideal for evaluating our setup’s mechanical precision and imaging reliability. The gantry achieved high angular precision with a fixed 2°step resolution, enabling consistent and reproducible imaging across different orientations. Unlike conventional robotic US systems that offer 6–7 degrees of freedom (DoF) [33] and sub-millimetre precision but suffer from high cost ($1000–35000) and operational complexity [34], our low-cost system ($88) achieves sufficient spatial resolution for ex vivo scanning. Angular sweeps are completed within 0.1–0.5 s per step and take 4–5 min to scan the whole organ, with commercial robotic systems requiring 7–12 min per full scan. Timing may vary depending on the organ size and shape [35]. We used water immersion as the coupling medium to ensure uniform acoustic transmission during imaging. Phantoms were placed in a custom water-filled container and aligned with the scanning head of the gantry to maintain consistent probe–tissue interaction across angular views. This setup helped to maintain consistent acoustic transmission, eliminate air gaps, reduce image variability due to pressure inconsistencies and provided stable, high-quality B-mode US frames for training and testing. The CAE blue phantom, representing a breast tumor model, was used to validate the gantry’s effectiveness, as shown in Fig. 11. This phantom contained two tumors: tumor 1, positioned along the central scanning line, and tumor 2, located off to one side. When scanned using a linear gantry system [13], tumor 1 was scanned, and the optimal tumor slice algorithm was applied to predict the best slice location for microscopic analysis. This process resulted in a probability (p) of 0.97. However, tumor 2 could not be analyzed (p = 0) due to the linear gantry’s restricted, unidirectional scanning path, which limits coverage to a single plane. This restriction significantly hinders optimal tumor slice selection in cases where anatomical structures extend beyond the immediate scanning field. An automated angular gantry system was designed to address this limitation, allowing for angular scanning covering a broader phantom area. The angular gantry successfully scanned both tumors, and the optimal tumor slice algorithm was applied to predict the best slice location for pathological examination. Tumor 1 achieved an improved probability of 0.98, while tumor 2, previously undetected, was now recognized with a probability of 0.96. The overall accuracy increased to 98%, demonstrating the superior capability of the angular approach in obtaining a complete representation of the tumor structure.

**Table 4:** Performance comparison of various models. We used 800 US scans to test the models.

Network	Precision	Recall	F1-score	Accuracy
AlexNet [16]	0.80	0.80	0.79	0.80
Vgg 16	0.84	0.84	0.83	0.84
Vgg19 [17]	0.84	0.83	0.83	0.84
ResNet18	0.85	0.82	0.82	0.82
ResNet50 [18]	0.88	0.86	0.86	0.86
MobileNet [19]	0.88	0.88	0.88	0.88
DenseNet [20]	0.95	0.95	0.95	0.95
ConvNeXt [21]	0.92	0.91	0.91	0.92
DeiT [22]	0.98	0.97	0.96	0.97
Swin [23]	**0.99**	**0.97**	**0.98**	**0.98**

### 8.1 Model performance

**Fig. 12:**
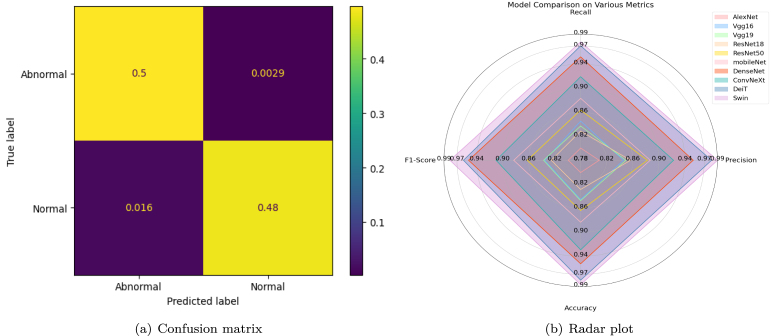
(a) Confusion matrix of Swin transformer, showing the performance of the trained model by illustrating the number of correct and incorrect predictions for each class. (b) presents a radar plot that compares various performance metrics across multiple models.

A total of 4900 B-mode US images derived from two anatomically distinct phantoms were used in this study. One phantom was used for training, while the other was reserved for testing to ensure domain-level separation. Deep learning architectures were benchmarked, including CNNs, lightweight models (MobileNet), and transformer-based networks. The key training and architectural parameters, including input image size, number of trainable parameters, floating-point operations per second (FLOPs), and inference throughput, are provided in Table 3, which summarizes the comparative deployment efficiency and computational cost of all evaluated models. Testing was conducted on the GPU, achieving inference times of 35–45ms per frame for the Swin Transformer. These metrics highlight the model’s readiness for real-time use. Swin Transformer achieved the best results, with a precision of 0.99, recall of 0.97, F1-score of 0.98, and accuracy of 0.98. Confusion matrices and radar charts were utilized to compare model performance across various metrics visually. The radar plot (Fig. 12(b)) facilitated a comparative analysis of model precision, recall, F1-score, and accuracy. In contrast, the confusion matrix (Fig. 12(a)) demonstrated the effectiveness of the automated hardware-assisted algorithms in identifying optimal slice locations by scanning tumor phantoms in real-time. These quantitative experimental results are consistent with previously reported deep learning-based tumor classification methods, which have shown robust performance [36], [37].

### 8.2 Interpretability and workflow efficiency

**Fig. 13:**
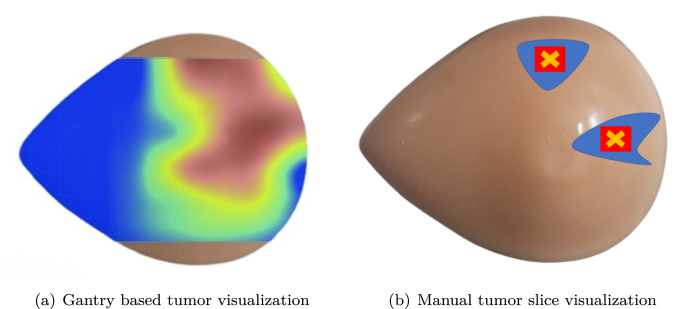
Comparison of slice prediction using the angular ultrasound gantry (a) and manual method (b). The gantry provides tumor probability for each slice, aiding accurate tissue selection, while manual prediction is time-consuming, error-prone, and lacks quantitative guidance.

**Fig. 14:**
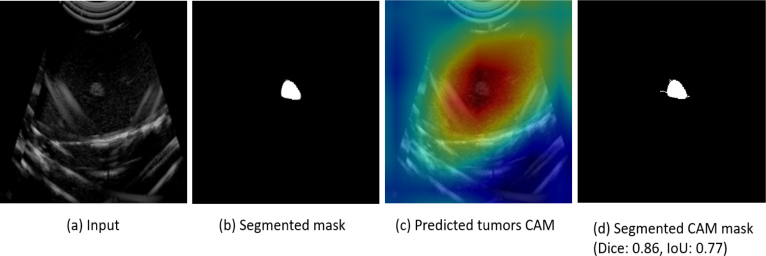
Comparison between the ground truth tumor mask (b) and the predicted tumor region segmented from the class activation map (d) for a breast ultrasound image. The predicted mask shows strong alignment with the annotated ground truth, achieving a Dice coefficient of 0.86 and an IoU of 0.77.

We employed CAMs to visualize the discriminative image regions that contributed most to the model’s prediction. These CAMs overlay heatmaps on original B-mode scans to identify tumor-relevant structures. A trained pathologist reviewed these outputs, confirming that the highlighted regions consistently aligned with known tumor locations. Fig. 13 shows a visualization of manual slice selection with automated CAM-based predictions. Manual selection often required multiple cuts and subjective judgment, increasing the risk of suboptimal sampling. In contrast, the gantry-guided CAM predictions were consistent and reproducible, reducing error and expediting tissue triage. Although the validation is primarily qualitative and quantitative, Fig. 14 shows the Dice and IoU for the input mask and the predicted. The segmented tumor region derived from the CAM closely matches the ground truth annotation, achieving a Dice coefficient of 0.86 and an IoU of 0.77, reinforcing the prediction’s clinical relevance and spatial accuracy.

### 8.3 Operational and clinical relevance

**Fig. 15:**
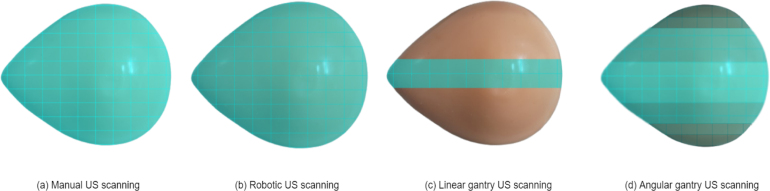
(a) the phantom coverage achieved during manual ultrasound scanning highlights inconsistent and limited area capture. (b) displays improved but constrained coverage using a robotic arm. (c) illustrates the uniform linear coverage obtained using a linear gantry system. (d) presents comprehensive and angularly diverse phantom coverage enabled by the proposed gimbal-based gantry system. Last phantoms most top and bottom part dark because it is not cover in scanning.

The gantry system was designed for post-surgical or ex vivo tissue processing environments. Its integration of mechanical motion and deep learning-based inference bridges the gap between manual and high-cost robotic tissue analysis. The current system covers approximately 90% of the tumor mass during scanning. Traditional manual selection of tissue slices is time-intensive and prone to human error. By automating this process, the gantry minimizes errors and decreases the time and resources necessary to determine the most diagnostically relevant slices. The hardware’s portability and remote operation make it ideal for rural or underserved areas with limited pathologist access. Manual scanning is operator-dependent, time-consuming, and inconsistent coverage due to variability in probe handling. While offering some automation, robotic arms are prohibitively expensive and complex to operate, making them less feasible for widespread or low-resource deployment. In contrast, the proposed gimbal-based gantry system provides a low-cost, automated, consistent scanning solution that ensures comprehensive and uniform phantom coverage. The coverage area of the phantom using different methods such as manual, robotic, and gimbal is shown in Fig. 15. The gantry can be deployed in various medical centers, institutions, and hospitals without requiring specialized training, making it an accessible tool for researchers. Beyond model performance, broader considerations for clinical adoption must be addressed. These include tissue preservation protocols, biosafety handling, and regulatory compliance. Our current setup, is based on phantom data in future we plan to expand the dataset using additional phantoms with anatomical variation and, eventually, real human tissue samples.

### 8.4 Limitations and future directions

The phantom-based validation provides a reproducible setting for algorithm and hardware assessment; however, it lacks biological variability in real tissues. Although CAE Blue phantoms simulate soft-tissue acoustics, they cannot fully replicate heterogeneity in fat, fibrosis, or tumor margins [38], [39], [40]. To address this, future validation will incorporate anatomically diverse phantoms and ethically sourced clinical tissue samples to assess model generalizability. Currently, the system supports only B-mode ultrasound and achieves 90% coverage of the tumor mass. Upcoming versions aim to integrate multimodal imaging, such as Doppler and elastography, and extend scanning coverage to near-complete levels. Advanced preprocessing pipelines and heterogeneous embedded systems will also be explored to improve robustness and real-time efficiency without increasing latency [30], [31], [41], [42], [43]. Additional work will focus on long-term mechanical reliability through closed-loop calibration and alignment protocols. Concurrently, efforts are underway to meet biosafety and regulatory requirements for future clinical deployment.

## 9 Conclusion and future work

This paper presents the design and implementation of an angular US gantry system to enhance optimal tissue slice selection for pathology. This low-cost, precision-controlled hardware framework enables multi-angular scanning of excised tumor masses, ensuring comprehensive tissue characterization. Integrated with deep learning algorithms, the system assists in selecting the most diagnostically relevant tissue slices, significantly improving accuracy and efficiency over traditional manual methods. Experimental results demonstrate the system’s ability to acquire B-mode US images and deliver optimal tissue slice for microscopic analysis. However, the current setup is limited by the size and mobility of the gantry. In the future, we can focus on refining deep learning algorithms to achieve higher precision in tissue characterization, expand the coverage of full tumor mass scanning, and include a wider range of pathologies. Additionally, design limitations identified during the current phase can be addressed to enhance the functionality and effectiveness of the gantry system.

## CRediT authorship contribution statement

**Abhishek Kumar:** Writing – original draft, Visualization, Methodology, Formal analysis, Conceptualization. **Akshay S. Menon:** Methodology, Data curation. **Divyansh Sharma:** Resources. **Raviteja Sista:** Writing – review & editing, Methodology. **Debdoot Sheet:** Writing – review & editing, Supervision, Conceptualization.

## Ethics statements

The work does not use any human or animal subjects.

## Declaration of competing interest

The authors declare that they have no known competing financial interests or personal relationships that could have appeared to influence the work reported in this paper.

## Appendix A Supplementary data

Video S1 The supplementary materials contain three videos showing gantry header assembly, full gantry assembly, and a real-time automated ultrasound scan.

## Footnotes

1 https://amzn.in/d/dzHtATW.

2 https://robu.in/product/towerpro-sg90-9gm-1-2~kg-180-degree-rotation-servo-motor-good-quality/.

3 https://amzn.in/d/hHamToq.

4 https://amzn.in/d/6W6tAnA.

5 https://amzn.in/d/c4hFapD.

6 https://amzn.in/d/a0cBYAH.

7 https://amzn.in/d/8gN8CgR.

8 https://amzn.in/d/4wi1VKM.

9 https://shorturl.at/h6uVP.

10 https://www.creality.com/products/creality-ender-3-v3-se.

11 http://surabi.com/ethiroliTiny16a.html.

12 https://www.optisafe.se/blue-phantom-elastography-ultrasound-breast-phantom.

13 https://amzn.in/d/4C28AlV.
